# Characterization of a Cobalt-Substituted Globin-Coupled Oxygen Sensor Histidine Kinase from *Anaeromyxobacter* sp. Fw109-5: Insights into Catalytic Regulation by Its Heme Coordination Structure

**DOI:** 10.1021/acsomega.1c05564

**Published:** 2021-12-06

**Authors:** Kenichi Kitanishi, Motoyuki Shimonaka, Masaki Unno

**Affiliations:** † Department of Chemistry, Faculty of Science, Tokyo University of Science, 1-3 Kagurazaka, Shinjuku-ku, Tokyo 162-8601, Japan; ‡ Graduate School of Science and Engineering, Ibaraki University, 4-12-1 Nakanarusawa, Hitachi, Ibaraki 316-8511, Japan; § Frontier Research Center for Applied Atomic Sciences, Ibaraki University, 162-1 Shirakata, Tokai, Naka, Ibaraki 319-1106, Japan

## Abstract

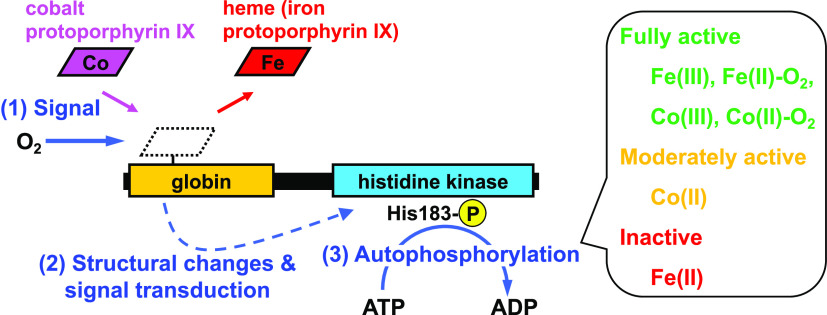

Heme-based gas sensors are an emerging class of heme proteins. *Af*GcHK, a globin-coupled histidine kinase from *Anaeromyxobacter* sp. Fw109-5, is an oxygen sensor enzyme in which oxygen binding to Fe(II) heme in the globin sensor domain substantially enhances its autophosphorylation activity. Here, we reconstituted *Af*GcHK with cobalt protoporphyrin IX (Co-*Af*GcHK) in place of heme (Fe-*Af*GcHK) and characterized the spectral and catalytic properties of the full-length proteins. Spectroscopic analyses indicated that Co(III) and Co(II)-O_2_ complexes were in a 6-coordinated low-spin state in Co-*Af*GcHK, like Fe(III) and Fe(II)-O_2_ complexes of Fe-*Af*GcHK. Although both Fe(II) and Co(II) complexes were in a 5-coordinated state, Fe(II) and Co(II) complexes were in high-spin and low-spin states, respectively. The autophosphorylation activity of Co(III) and Co(II)-O_2_ complexes of Co-*Af*GcHK was fully active, whereas that of the Co(II) complex was moderately active. This contrasts with Fe-*Af*GcHK, where Fe(III) and Fe(II)-O_2_ complexes were fully active and the Fe(II) complex was inactive. Collectively, activity data and coordination structures of Fe-*Af*GcHK and Co-*Af*GcHK indicate that all fully active forms were in a 6-coordinated low-spin state, whereas the inactive form was in a 5-coordinated high-spin state. The 5-coordinated low-spin complex was moderately active—a novel finding of this study. These results suggest that the catalytic activity of *Af*GcHK is regulated by its heme coordination structure, especially the spin state of its heme iron. Our study presents the first successful preparation and characterization of a cobalt-substituted globin-coupled oxygen sensor enzyme and may lead to a better understanding of the molecular mechanisms of catalytic regulation in this family.

## Introduction

Heme (iron protoporphyrin IX) is one of the best-known and most important cofactors required for proper biological functioning of many proteins and enzymes,^1^ including myoglobin (oxygen storage), hemoglobin (oxygen transfer), cytochrome *c* (electron transfer), cytochrome P450, and nitric oxide synthase (oxygen activation), among others.^1−4^

Heme also functions as the site for sensing gaseous molecules, including O_2_, NO, and CO, in heme-based gas sensor proteins.^3−6^ Generally, heme-based gas sensor proteins are composed of a heme-bound gas sensor domain at the N-terminus and a functional domain at the C-terminus. Association/dissociation of gaseous molecules to/from the heme iron induces structural changes in the sensor domain. These structural changes are then transduced to the functional domain, thereby switching on/off transcription or catalytic reactions.^3−7^ Globin-coupled oxygen sensors constitute an important family of oxygen sensor proteins in which the heme-bound sensor domain contains a globin fold similar to those of myoglobin and hemoglobin.^7−9^

Among globin-coupled oxygen sensors characterized to date, the globin-coupled histidine kinase from *Anaeromyxobacter* sp. Fw109-5, *Af*GcHK, has been the best studied from both structural and functional standpoints. *Af*GcHK is part of a two-component signal transduction system in an anaerobic, metal-reducing bacterium. *Af*GcHK consists of an N-terminal heme-bound globin sensor domain and a C-terminal histidine kinase domain. Oxygen binding to the Fe(II) heme in the sensor domain substantially enhances autophosphorylation at His183 using ATP in the kinase domain, after which the phosphoryl group is transferred to its cognate response regulator protein.^10^ More recently, a homologous protein from the closely related myxobacterial species, *Myxococcus xanthus*, was reported to be involved in motility through the expression of pilus genes.^11^ Currently, increasing numbers of genes encoding orthologous proteins are being found in many bacterial genomes.

In our previous studies, we characterized the spectroscopic and catalytic properties of *Af*GcHK,^10,12^ reporting the following findings: (1) the 6-coordinated low-spin (6cLS) Fe(III) and Fe(II)-O_2_, and Fe(II)-CO complexes of *Af*GcHK are active histidine kinase enzymes, whereas the 5-coordinated high-spin (5cHS) Fe(II) complex is inactive; (2) His99 is the heme axial ligand at the proximal side; (3) Tyr45 at the distal side is important for O_2_ recognition; (4) the Fe(II)-O_2_ complex is unusually stable (>3 days at room temperature); and (5) oxygen binding to the heme and redox changes in the heme of the globin domain modulate substrate (ATP) affinity and catalytic activity in the functional domain.

Although crystal structures of the isolated globin domain of *Af*GcHK in cyanide-liganded [Fe(III)-CN] and partially unliganded [mixture of Fe(III)-CN and Fe(II)] forms have been determined,^13^ the molecular mechanism of catalytic regulation by O_2_ binding to the Fe(II) heme complex is not yet fully understood. Heme replacement with another metalloporphyrin or a porphyrin with different peripheral side chains is a direct and powerful approach for elucidating the role of heme in proteins.^14^ Notable in this context, there have been no reports of metal-substituted globin-coupled sensors to date. Using this substitution approach, we further investigated the molecular mechanism of the catalytic regulation of *Af*GcHK. To this end, we reconstituted *Af*GcHK with cobalt protoporphyrin IX as a model of a globin-coupled oxygen sensor and explored its structure–function relationships by examining its spectral and catalytic properties using optical absorption spectroscopy and enzymatic assays. We propose that the catalytic activity of *Af*GcHK is regulated by its heme coordination structure, especially the spin state of its heme iron.

## Results

In previous studies, *Af*GcHK was expressed in *Escherichia coli*, reconstituted with heme by adding heme to the crude extract after disrupting *E. coli* cells by sonication, and purified as heme-bound form (hereafter referred to as Fe-*Af*GcHK).^10,12^ Even adding the heme precursor, 5-aminolevulinic acid, to the growth medium upon inducing protein expression did not result in heme incorporation into the heme-binding site of the target protein inside *E. coli* cells. Using this system, we reconstituted *Af*GcHK with cobalt protoporphyrin IX (hereafter referred to as Co-*Af*GcHK) and characterized it, comparing differences in its spectroscopic and catalytic properties with those of Fe-*Af*GcHK. It should be noted that heme and cobalt protoporphyrin IX share the same porphyrin ring structure, differing only in terms of the metal in the central position. In addition, O_2_ can bind to both Fe(II) and Co(II) states of heme and cobalt porphyrin, respectively, but CO can bind only to the Fe(II) state of heme and not to the Co(II) state of cobalt porphyrin.^15^

### Purification of Fe-*Af*GcHK and Co-*Af*GcHK

**Figure 1:**
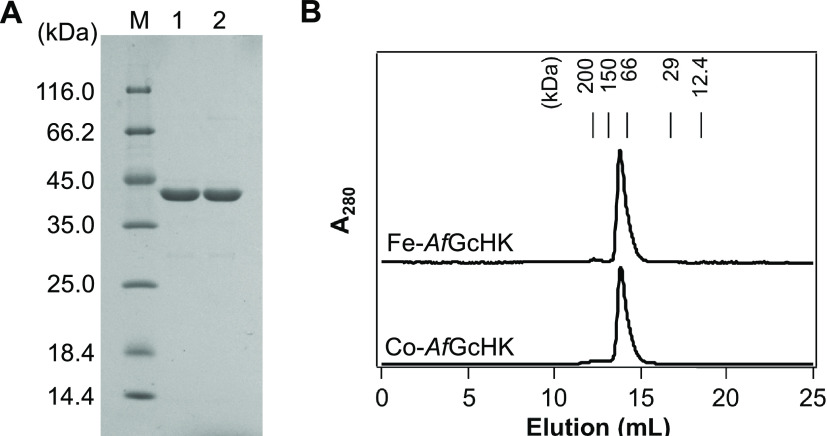
Purification of Fe-*Af*GcHK and Co-*Af*GcHK. (A) Purity of Fe-*Af*GcHK and Co-*Af*GcHK determined by SDS-PAGE analysis using 12% gels. Molecular mass markers, denoted by M, are shown in the left lane. Lane 1, Fe-*Af*GcHK; lane 2, Co-*Af*GcHK. (B) Elution profiles of Fe-*Af*GcHK and Co-*Af*GcHK on a gel filtration column reveal that both proteins behave as dimers. Molecular mass markers are shown at the top.

Affinity and gel filtration column chromatography techniques were used to purify full-length Fe-*Af*GcHK and Co-*Af*GcHK proteins. The purity of the resulting proteins was judged to be >90%, as confirmed by SDS-PAGE analysis. The single band observed on SDS-PAGE gels corresponded to the predicted mass of 43.0 kDa for the full-length protein with a C-terminal His_6_ tag (Figure 1A). As previously reported for Fe-*Af*GcHK,^10^ both purified Fe-*Af*GcHK and Co-*Af*GcHK eluted as single peaks by analytical gel filtration chromatography with a molecular mass of 90 kDa, consistent with a homodimeric form (Figure 1B).

### Metal Content of Fe-*Af*GcHK and Co-*Af*GcHK

The metal contents of purified Fe-*Af*GcHK and Co-*Af*GcHK were quantified by inductively coupled plasma optical emission spectroscopy (ICP-OES). Fe-*Af*GcHK contained one equivalent of iron, indicating that Fe-*Af*GcHK contains one equivalent of heme iron, as previously confirmed using the pyridine hemochromogen method.^10^ Similarly, Co-*Af*GcHK contained one equivalent of cobalt, without any detectable iron, indicating that Co-*Af*GcHK contains one equivalent of cobalt protoporphyrin IX instead of heme. Thus, we successfully prepared Co-*Af*GcHK.

### Far-UV Circular Dichroism (CD) Spectra of Fe-*Af*GcHK and Co-*Af*GcHK

**Figure 2:**
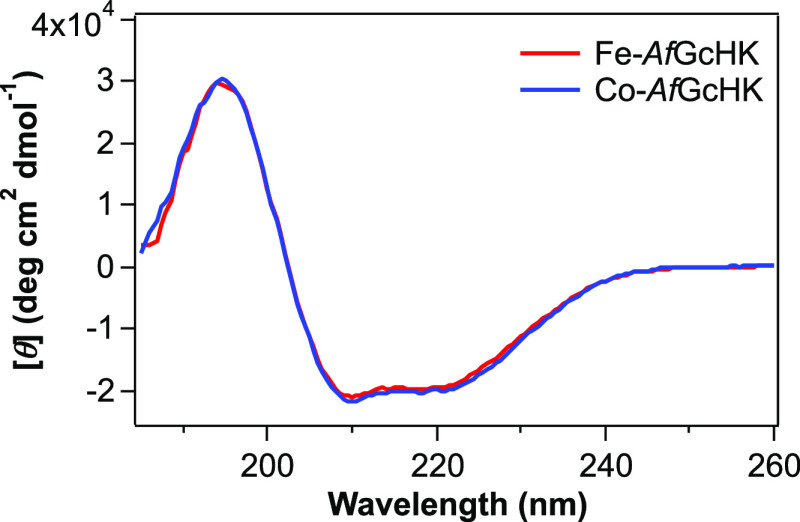
Far-UV CD spectra of Fe-*Af*GcHK (red line) and Co-*Af*GcHK (blue line). Protein concentration was 20 μM, and the buffer was 20 mM Tris-HCl, pH 8.0, 50 mM NaCl.

To compare the secondary structure of *Af*GcHK between Fe-*Af*GcHK and Co-*Af*GcHK, we measured far-UV CD spectra. The CD spectra of both Fe-*Af*GcHK and Co-*Af*GcHK exhibited minima at 210 and 221 nm, indicative of a primarily helical structure (Figure 2), a finding consistent with the crystal structures of the isolated globin domain of *Af*GcHK and homology models of the full-length protein constructed in previous studies.^13,16^ The similarity between the CD spectra of Fe-*Af*GcHK and Co-*Af*GcHK suggests that the difference in the central metal of the porphyrin cofactor does not induce a change in the overall helical secondary structure content or cause major structural alterations.

### Optical Absorption Spectra of Fe-*Af*GcHK and Co-*Af*GcHK

**Figure 3:**
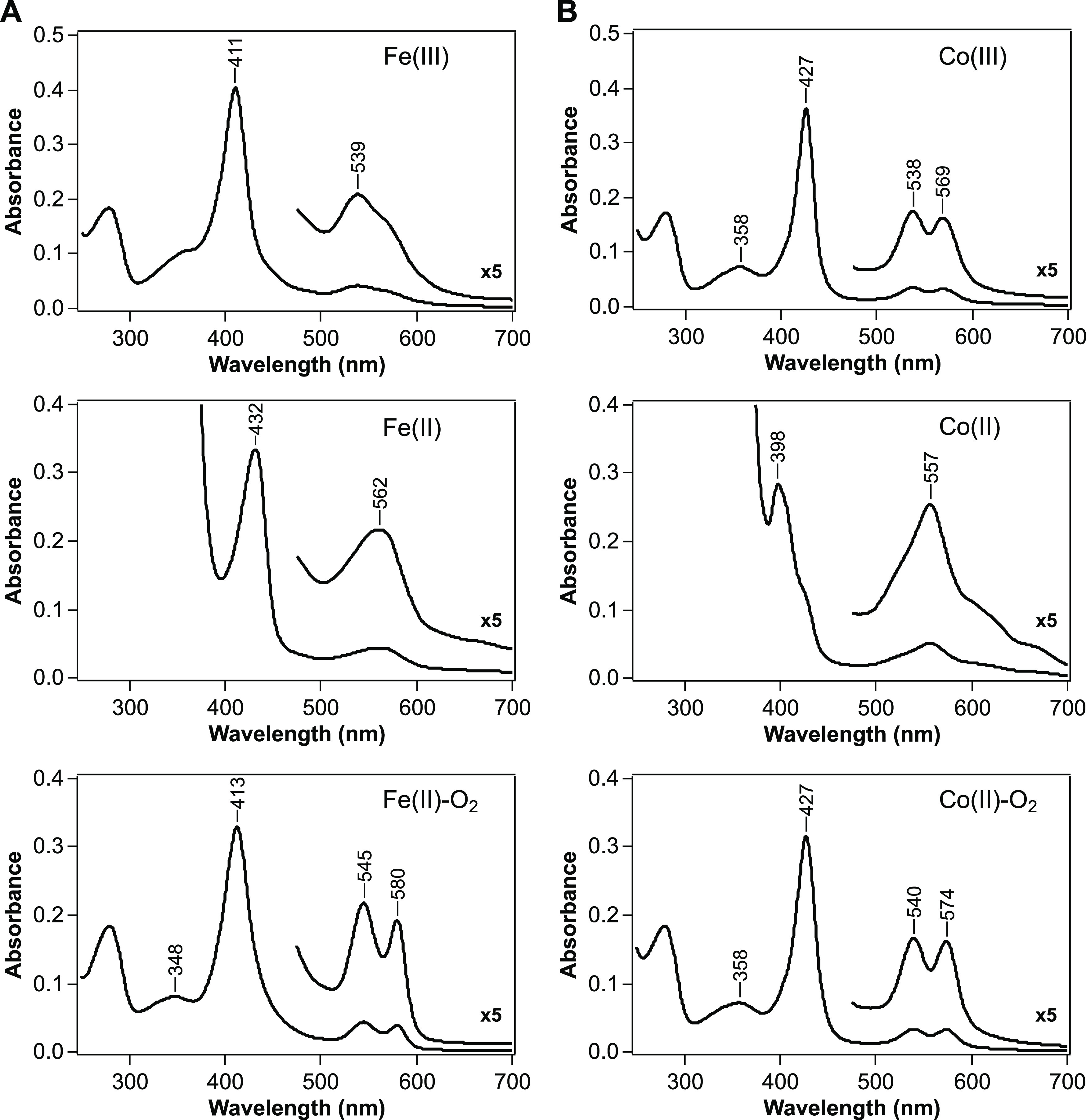
Absorption spectra of oxidized [Fe(III) and Co(III); top], reduced [Fe(II) and Co(II); middle], and oxygen-bound [Fe(II)-O_2_ and Co(II)-O_2_; bottom] forms of (A) Fe-*Af*GcHK and (B) Co-*Af*GcHK. Protein concentration was 4 μM, and the buffer was 50 mM Tris-HCl, pH 8.0, 100 mM NaCl. The visible region of the spectrum (475–700 nm) has been enlarged 5-fold. The absorption maxima of these proteins are summarized in Table 1.

Optical absorption spectra were collected for oxidized [Fe(III) and Co(III)], reduced [Fe(II) and Co(II)], and oxygen-bound [Fe(II)-O_2_ and Co(II)-O_2_] forms of Fe-*Af*GcHK and Co-*Af*GcHK (Figure 3). The absorption maxima of Fe-*Af*GcHK and Co-*Af*GcHK are summarized in Table 1.

**Table 1:** Absorption Spectra of Oxidized [M(III)], Reduced [M(II)], and Oxygen-Bound [M(II)-O_2_] forms of Fe-*Af*GcHK and Co-*Af*GcHK. M = Fe or Co^a^

	M(III)	M(II)	M(II)-O_2_
**Heme Proteins**			
Fe-*Af*GcHK	411, 539	432, 562	348, 413, 545, 580
	His/OH^–^	His	His/O_2_
	6cLS	5cHS	6cLS
Mb^b^	358, 414, 542, 582^e^	434, 556	348, 418, 543, 581
	His/OH^–^	His	His/O_2_
	6cLS	5cHS	6cLS
Hb^b^	410, 540, 575^e^	430, 555	344, 415, 541, 577
	His/OH^–^	His	His/O_2_
	6cLS	5cHS	6cLS
**Cobalt-Substituted Proteins**			
Co-*Af*GcHK	358, 427, 538, 569	398, 557	358, 427, 540, 574
	His/OH^–^	His	His/O_2_
	6cLS	5cLS	6cLS
CoHRP^c^	427, 538, 572^e^	401, 553	424, 535, 567
	His/OH^–^	His	His/O_2_
	6cLS	5cLS	6cLS
CoMb^d^	not reported	406, 558	426, 539, 577
		His	His/O_2_
		5cLS	6cLS
CoHb^d^	not reported	402, 552	428, 538, 571
		His	His/O_2_
		5cLS	6cLS

a Corresponding spectra of other relevant native and cobalt-substituted heme proteins are shown as a reference. Proposed coordination structures are also presented. 6cLS, 6-coordinated low-spin; 5cHS, 5-coordinated high-spin; 5cLS, 5-coordinated low-spin.

b Reference (2).

c Reference (17).

d Reference (18).

e For comparison with *Af*GcHK, alkaline OH^–^ forms are shown.

The Soret band of the Co(III) complex of Co-*Af*GcHK was red-shifted by 16 nm (to 427 nm) relative to that of the Fe(III) complex of Fe-*Af*GcHK (411 nm) (Figure 3 and Table 1). The visible region in the spectrum of Co-*Af*GcHK revealed two well-resolved α and β peaks at 569 and 538 nm, respectively, which contrasts with the broad absorption of Fe-*Af*GcHK at ∼539 nm and shoulder at ∼570 nm (Figure 3 and Table 1). The absorption spectrum of the Co(III) complex of Co-*Af*GcHK also displayed a distinct δ band at 358 nm, which is not clearly detectable in Fe-*Af*GcHK (Figure 3 and Table 1). Based on similarity with the absorption spectrum of cobalt-substituted horseradish peroxidase (CoHRP) at alkaline pH (pH > 9.5) (Table 1),^17^ this spectrum was assignable to a 6-coordinated low-spin (6cLS) state, and OH^-^ was suggested to be the sixth ligand trans to the fifth axial ligand, His99, which is similar to the Fe(III) complex of Fe-*Af*GcHK.^10^

Even the addition of sodium dithionite did not reduce the Co(III) complex of Co-*Af*GcHK (data not shown); similar observations have been reported for cobalt-substituted myoglobin (CoMb) and hemoglobin (CoHb).^18^ This is unlike the case for Fe-*Af*GcHK, which was easily reduced by adding sodium dithionite, which shifted the Soret band to 432 nm from 411 nm, and caused α and β bands to merge into a single band (562 nm) (Figure 3 and Table 1). However, in the presence of methyl viologen, an electron mediator, the Co(III) complex of Co-*Af*GcHK was reduced efficiently to the Co(II) complex; the Soret band was shifted to shorter wavelengths (398 nm) from 427 nm rather than to longer wavelengths, and the α and β bands merged into a single band (557 nm) (Figure 3 and Table 1), which was assigned to a 5-coordinated low-spin (5cLS) state. It should be noted that both the Co(III) and Co(II) atoms of cobalt porphyrin are low-spin, regardless of the oxidation state.^19^

The absorption spectrum of the Co(II)-O_2_ complex of Co-*Af*GcHK was almost identical to that of the Co(III) complex of Co-*Af*GcHK (Figure 3), except for a slight decrease in Soret band extinction and slight changes in visible regions, as also previously reported for CoMb and CoHb.^18,20^ Notably, the Co(II)-O_2_ complex of Co-*Af*GcHK was easily reduced to the Co(II) complex within ∼5 min by adding only dithionite, even in the absence of methyl viologen, but the Co(III) complex of Co-*Af*GcHK was not reduced by dithionite alone. Furthermore, unlike Fe-*Af*GcHK, which shifts from high-spin to low-spin upon oxygen binding, Co-*Af*GcHK remained in a low-spin state. All of these spectral properties are similar to those previously reported for CoMb and CoHb (Table 1).^15,18,20^

### Catalytic Activities of Fe-*Af*GcHK and Co-*Af*GcHK

**Figure 4:**
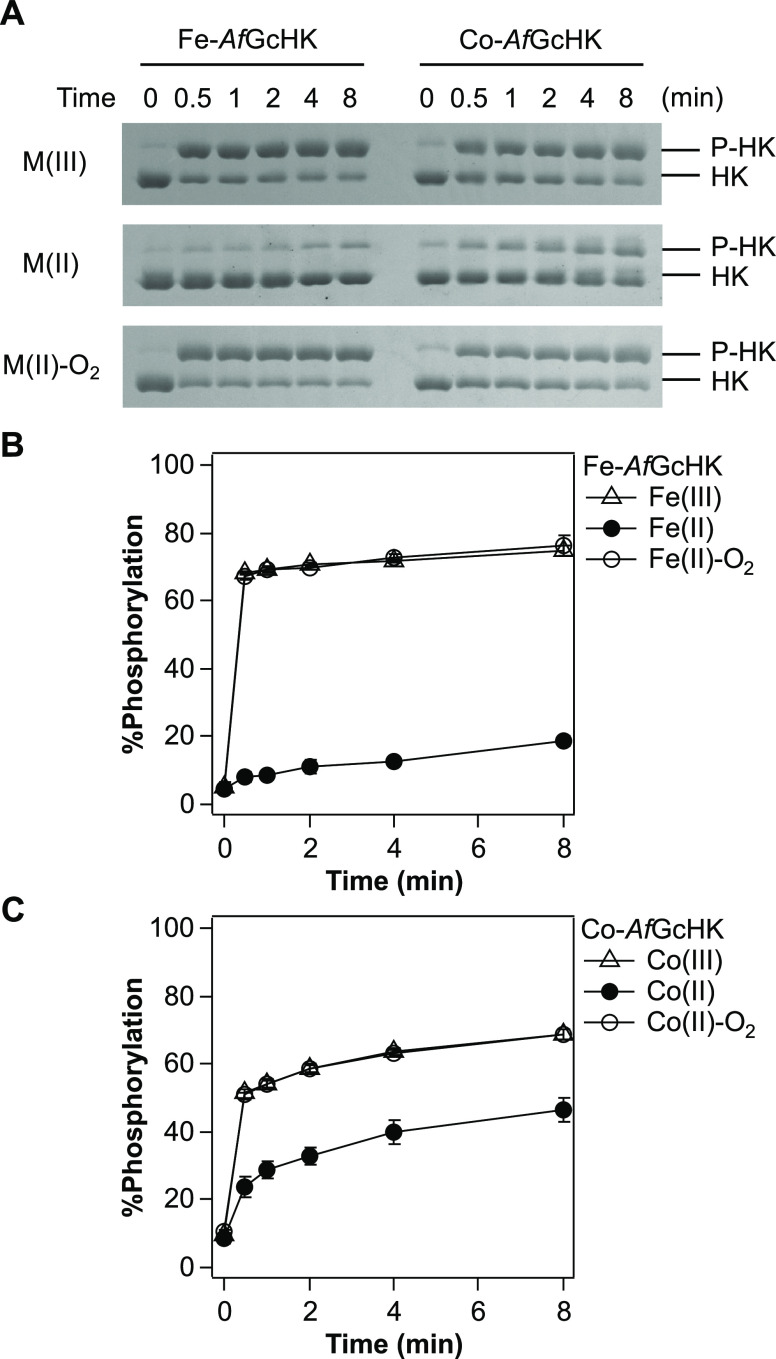
Autophosphorylation activities of Fe-*Af*GcHK and Co-*Af*GcHK. (A) Phos-tag SDS-PAGE gel patterns demonstrate a time-dependent increase in phosphorylated *Af*GcHK (upper band, P-HK) and a simultaneous decrease in nonphosphorylated *Af*GcHK (lower band, HK) catalyzed by the various complexes of Fe-*Af*GcHK and Co-*Af*GcHK. Data were obtained at the indicated times after initiation of the reaction. (B, C) Time-courses of autophosphorylation of (B) Fe-*Af*GcHK and (C) Co-*Af*GcHK for oxidized [M(III); open triangles], reduced [M(II); closed circles], and oxygen-bound [M(II)-O_2_; open circles] forms. M = Fe or Co. Data are presented as means ± S.D. of at least three independent experiments.

We examined the autophosphorylation activities of various iron and cobalt complexes of *Af*GcHK using Phos-tag SDS-PAGE, which differentiates between nonphosphorylated and phosphorylated proteins (Figure 4). Previous studies have shown that the catalytic reaction is rapid and almost completed within 5 min at 25 °C.^10,12^ Additionally, the as-purified sample was partially pre-autophosphorylated (∼10%), and the degree of pre-autophosphorylation, which probably occurred during expression and purification stages, was variable between preparations.^10^ Because it was difficult to determine precise kinetic parameters for autophosphorylation activity, we categorized the catalytic activity into three groups: fully active, moderately active, and inactive.

Previous studies indicated that the Fe(III), Fe(II)-O_2_, and Fe(II)-CO complexes of Fe-*Af*GcHK clearly display autophosphorylation activity, whereas the Fe(II) complex does not.^10,12^ Consistent with these previous results, the Fe(III), and Fe(II)-O_2_ complexes of Fe-*Af*GcHK displayed autophosphorylation activity, and the proportion of autophosphorylated protein reached a maximum of ∼75% at 8 min; in contrast, the maximum reached by the Fe(II) complex was ∼20% (Figure 4A,B). Thus, Fe(III) and Fe(II)-O_2_ complexes are fully active forms, whereas the Fe(II) complex is an inactive form.

Co(III) and Co(II)-O_2_ complexes of Co-*Af*GcHK displayed a similar autophosphorylation activity (∼70%) (Figure 4A,C) compared with Fe(III) and Fe(II)-O_2_ complexes of Fe-*Af*GcHK, suggesting that the central metal does not significantly affect catalytic activity. These forms were grouped into “fully active”. Unexpectedly, the Co(II) complex of Co-*Af*GcHK exhibited slightly less but sufficient autophosphorylation activity (∼50%) compared with Co(III) and Co(II)-O_2_ complexes (Figure 4A,C) and was categorized as a “moderately active” form, distinguishing it from the inactive Fe(II) complex of Fe-*Af*GcHK.

Collectively, these findings indicate that all fully active complexes—Fe(III), Co(III), Fe(II)-O_2_, and Co(II)-O_2_—were 6cLS, whereas the inactive complex, Fe(II), was 5cHS. We also newly discovered that the 5cLS complex, Co(II), was a moderately active form. Therefore, these observations suggest that the coordination structure of the porphyrin cofactor in the globin sensor domain regulates the autophosphorylation activity of its functional domain.

## Discussion

Heme replacement with similar metalloporphyrin analogues is a powerful approach for understanding the function of heme in heme proteins. Reconstitution of apoprotein with non-iron metalloporphyrins has long been used in studies of heme-containing proteins ranging from typical hemoproteins such as myoglobin and hemoglobin to recently identified heme sensor proteins.^15,17−24^ Nevertheless, among globin-coupled oxygen sensors, no metal-substituted proteins have been reported prior to this study, which is the first report of a cobalt-substituted globin-coupled oxygen sensor enzyme.

Cobalt porphyrin has a unique electronic structure compared with that of heme. The Co(II) atom of cobalt porphyrin is low-spin (3d^7^, *S* = 1/2) in both oxy [Co(II)-O_2_] and deoxy [Co(II)] states, whereas the Fe(II) atom of heme changes from high-spin (3d^6^, *S* = 2) to low-spin (*S* = 0) upon oxygen binding.^19^ Because heme-based sensors often exert redox-dependent and/or ligand (gas)-dependent catalytic regulation, characterizing their cobalt-substituted protein can unveil molecular mechanisms hidden by the spin-state transition of the heme iron.

**Figure 5:**
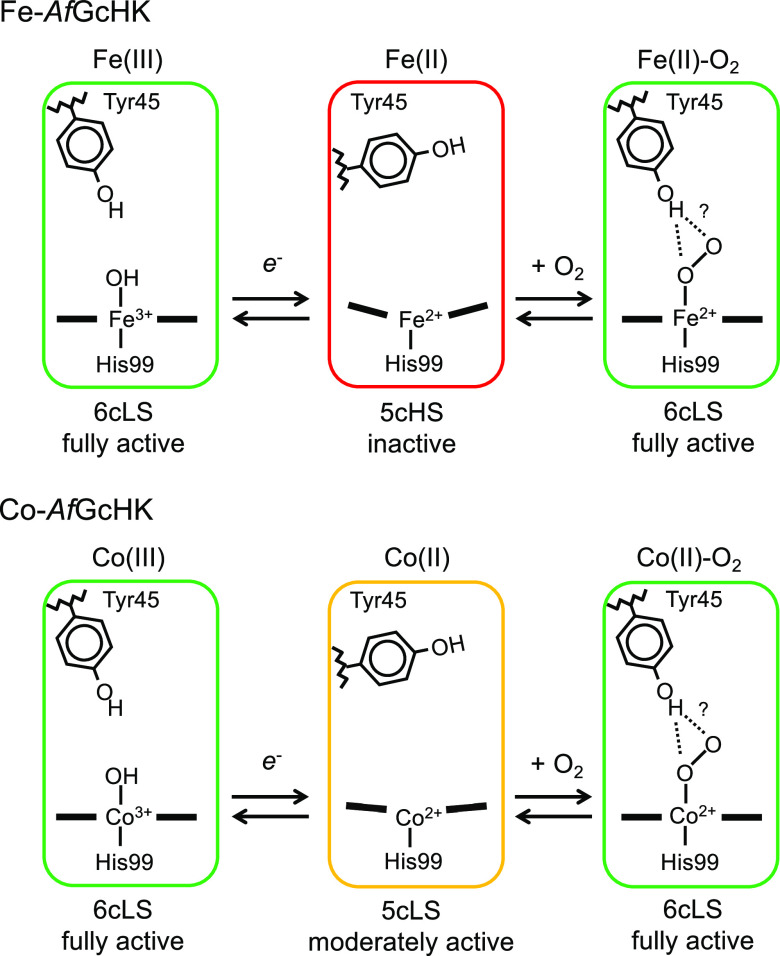
Proposed coordination structures of heme and cobalt porphyrin relevant to the catalytic activities of Fe-*Af*GcHK and Co-*Af*GcHK, respectively. The 6cLS complexes [Fe(III), Fe(II)-O_2_, Co(III), and Co(II)-O_2_] are fully active forms, the 5cHS complex [Fe(II)] is inactive, and the 5cLS complex [Co(II)] is moderately active, the latter of which is a novel finding of this study. By analogy with the Fe(II)-O_2_ complex of Fe-*Af*GcHK, Tyr45-OH is predicted to interact with the proximal O atom, but interaction(s) with the distal O atom cannot be totally ruled out for the Co(II)-O_2_ complex of Co-*Af*GcHK. Color codes are similar to those of traffic lights, with fully active shown in green, moderately active in yellow, and inactive in red.

In this study, we revealed that the catalytic activity of *Af*GcHK is regulated by the coordination structure, especially the spin state of its heme iron. In contrast to low-spin heme iron, which sits on the porphyrin plane, it is known that in high-spin heme, iron moves out of the porphyrin plane. Therefore, this catalytic regulation may be explained in terms of how far the metal is out of the porphyrin plane (i.e., the distance of the metal from the porphyrin plane), as discussed below and illustrated in Figure 5.

Although the crystal structures of some states of the isolated globin domain of *Af*GcHK have been determined,^13,25^ not all structures discussed here are currently available. Because of this, we speculate on the distance of the metal from the porphyrin plane in *Af*GcHK based on the structures of the corresponding myoglobin complexes.^19,26^ In the crystal structures of native and cobalt-substituted sperm whale myoglobin, distances of the metal from the porphyrin plane are 0.089–0.11 Å for 6cLS [Fe(II)-O_2_, Co(III)-H_2_O, and Co(II)-O_2_], 0.15 Å for 5cLS [Co(II)], and 0.39 Å for 5cHS [Fe(II)].^19,26^ Applying this trend to the case of *Af*GcHK yields an estimated order of 5cHS ≫ 5cLS > 6cLS complexes, which correspond to inactive, moderately active, and fully active forms, respectively, in terms of autophosphorylation activity, indicating a correlation between the heme coordination structure and catalytic activity. In tetrameric human hemoglobin, the movement of iron into and out of the porphyrin plane triggers an allosteric transition between the “tense (T) state” and the “relaxed (R) state”, which has been described as a driving force in cooperative oxygen binding. Although the evolutionary relationship between the vertebrate globin and bacterial globin-coupled sensor is currently unknown,^27^ it would be interesting if globin-coupled oxygen sensors also utilize a similar mechanism for signaling and switching on/off the activation of its functional domain.

In our previous work on *Af*GcHK^10^ and another globin-coupled oxygen sensor diguanylate cyclase from *E. coli*, YddV^28^ (also known as *Ec*DosC), we also suggested that the catalytic activity of globin-coupled oxygen sensors depends on the spin state. Our current findings further corroborate this concept through the characterization of a cobalt-substituted protein. Another example of spin-state-dependent catalytic regulation of a heme-based sensor enzyme is found in FixL, an oxygen sensor histidine kinase containing a heme-bound PAS domain. In FixL, catalysis also depends on the spin state of heme iron but not the oxidation state (i.e., high-spin Fe(III) and Fe(II): active form; low-spin Fe(II)-O_2_: inactive form).^29^ Thus, such spin-state-dependent catalytic regulation could be more universal than expected for heme-based sensors. However, not all heme-based gas sensors employ spin-state-dependent catalytic regulation. For example, the *E. coli* direct oxygen sensor, *Ec*DOS (also known as *Ec*DosP), displays a 6cLS complex with His77/Met95 axial ligation in the Fe(II) state; O_2_ replaces Met95 and binds to the heme iron and thereby activates the phosphodiesterase activity of the enzyme.^30^ Because the Fe(II)-O_2_ complex is also 6cLS, its spin state does not change upon oxygen binding.

Furthermore, this spin-state-dependent catalytic regulation may be also correlated with heme distortion, as was recently described for *Bpe*GReg,^31^ another globin-coupled oxygen sensor diguanylate cyclase from *Bordetella pertussis*, and bacterial heme-based NO sensor, H-NOX domain proteins.^32^ As is the case for *Af*GcHK, gas binding to the distorted 5cHS Fe(II) heme alleviates heme distortion in these sensors, leading to conformational changes in the heme-bound sensor domain and subsequent changes in intra- and/or intermolecular interactions with partner proteins and downstream signal transduction.^32^

Our study focused on the heme coordination structure as an initial signal that induces conformational changes in the globin sensor domain through ligand binding and/or redox changes, thereby propagating the signal to its functional domain. However, without structural information for the full-length protein, the mechanism underlying activation of the functional domain in response to oxygen binding to and/or a redox change in the heme iron of the globin sensor domain remains unclear at the atomic level. Clarifying this will require determining the structures of active (low-spin) and inactive (high-spin) *Af*GcHK. Nevertheless, in this study, we revealed the relationship between the heme coordination structure and catalytic activity, shedding light on the molecular mechanism of the catalytic regulation of *Af*GcHK, especially spin-state-dependent catalytic regulation. A recent hydrogen–deuterium exchange mass spectrometry (HDX-MS) study of full-length *Af*GcHK protein combined with the crystal structures of its isolated globin domain also indicated that striking structural changes at the heme proximal side are important in the signal transduction mechanism of *Af*GcHK,^13^ further supporting our current results.

## Conclusions

In this study, we prepared and characterized Co-*Af*GcHK in detail using optical absorption spectroscopy and enzymatic assays. Exploiting the unique properties of cobalt porphyrin, we revealed the relationship between the heme coordination structure and enzymatic activity. The 6cLS complexes of *Af*GcHK were fully active forms, whereas the 5cHS complex was an inactive form. We also newly discovered that the 5cLS complex is a moderately active form. To our knowledge, this is the first report describing a metal-substituted globin-coupled oxygen sensor enzyme and may provide insights that are applicable to other members of this family of globin-coupled oxygen sensor enzymes, a still emerging family of heme-based gas sensors. Collectively, our findings may lead to a better understanding of the molecular mechanism underlying the catalytic regulation of *Af*GcHK.

## Materials and Methods

### Materials

Cobalt(III) protoporphyrin IX chloride was purchased from Frontier Scientific (Logan, UT). Methyl viologen was purchased from Tokyo Chemical Industry (Tokyo, Japan). All other chemicals, acquired from FUJIFILM Wako Pure Chemical Corporation (Osaka, Japan) or Nacalai Tesque (Kyoto, Japan), were of the highest guaranteed grade available and were used without further purification.

### Expression and Purification of *Af*GcHK

*E. coli* BL21(DE3) (Novagen, Darmstadt, Germany) was transformed with a pET-21c vector expressing *Af*GcHK^10^ and grown overnight at 37 °C in 2.5 mL of Luria–Bertani medium (BD Difco) containing ampicillin (100 mg/L). Then, 0.5 L of the same medium containing ampicillin was inoculated with the starter culture (1:200 dilution) and grown at 37 °C. After 3 h, when the OD_600_ had reached 0.6–0.8, the temperature was reduced to 15 °C. Protein expression was induced by adding 0.1 mM isopropyl β-d-thiogalactopyranoside to the culture, and the cells were harvested by centrifugation 20 h later and cell pellets were stored at −80 °C until purification. Cell pellets (∼3 g from 0.5 L of culture) were suspended in 80 mL of Buffer A (50 mM Tris-HCl, pH 8.0, 100 mM NaCl) containing 1 mM phenylmethanesulfonyl fluoride. The cell suspension was stirred at 4 °C for 30 min and then sonicated (power setting, 5; duty, 50) on ice for 6 min at 2 min intervals (separated by 2 min cooling periods) using an ultrasonic disrupter (UD-201; TOMY SEIKO, Tokyo, Japan). The sonicate was centrifuged at 35 870*g* for 30 min, and the supernatant was incubated for 5 min with 50 μM hemin chloride or cobalt(III) protoporphyrin IX chloride in dimethyl sulfoxide solution and then loaded onto a HisTrap HP column (GE Healthcare) pre-equilibrated with Buffer A containing 20 mM imidazole. The column was washed with 100 mL of Buffer A containing 20 mM imidazole and eluted with 80 mL of a linear gradient from 20 to 300 mM imidazole in Buffer A. The fractions of interest were pooled and dialyzed overnight against 0.5 L of Buffer A. The dialyzed protein was concentrated to 5 mL using an Amicon Ultra-15 centrifugal filter device (Merck Millipore) and loaded onto a HiPrep 16/60 Sephacryl S-200 HR column (GE Healthcare) pre-equilibrated with Buffer A. The fractions of interest were pooled, concentrated, frozen in liquid nitrogen, and stored at −80 °C until further use. Protein concentrations were determined by Bradford protein assay using bovine serum albumin as a standard. *Af*GcHK is a homodimer, and its protein concentration is expressed in terms of subunit concentration throughout this study.

### Analytical Gel Filtration Chromatography

The oligomerization state of proteins was determined by gel filtration chromatography using the ÄKTAprime plus (GE Healthcare) chromatography system equipped with a Superdex 200 Increase 10/300 GL column (GE Healthcare). The buffer used for gel filtration chromatography was 50 mM Tris-HCl, pH 8.0, 100 mM NaCl. Molecular weight was estimated from the correlation between molecular weight and elution volume of standard proteins using a gel filtration molecular weight marker kit (Sigma-Aldrich, St. Louis, MO).

### Metal Content

Metal content was analyzed by ICP-OES using a SPECTRO ARCOS FHM22 system (SPECTRO Analytical Instruments, Kleve, Germany). Metal content was determined at commonly used analytical transitions of the atomic spectrum (Fe: 259.941, 239.562, and 238.204 nm; Co: 238.892, 230.786, and 228.616 nm). Standard curves for each metal were generated from dilutions of reference standard solutions prepared in 0.1 M nitric acid.

### Far-UV CD Spectra

CD spectra were recorded with a JASCO J-820 CD spectropolarimeter (Tokyo, Japan) using a demountable rectangular quartz cell (0.1 mm path length). Spectral data were collected four times at a bandwidth of 1 nm, a scan speed of 20 nm/min, and a response time of 4 s and combined.

### Optical Absorption Spectra

Absorption spectra were obtained using a V-630Bio (JASCO) spectrophotometer under aerobic conditions. Fe(II) and Co(II) complexes were prepared in N_2_-saturated buffer (50 mM Tris-HCl, pH 8.0, 100 mM NaCl) by adding sodium dithionite to the corresponding Fe(III) and Co(II)-O_2_ complexes. The N_2_-saturated solution was obtained by bubbling buffers with N_2_ gas for at least 30 min at room temperature. Fe(II)-O_2_ and Co(II)-O_2_ complexes were prepared by reducing Fe(III) and Co(III) complexes, respectively, with 10 mM sodium dithionite in the presence of 10 mM methyl viologen (only for Co-*Af*GcHK), after which excess dithionite and methyl viologen were removed by desalting using a Micro Bio-Spin 6 column (Bio-Rad Laboratories, Hercules, CA).

### Enzymatic Assays

Autophosphorylation activity was assayed at 25 °C in a reaction mixture containing 50 mM Tris-HCl, pH 8.0, 100 mM NaCl, 5 mM MgCl_2_, and 10 μM *Af*GcHK. The reaction mixture was preincubated for 5 min, and the reaction was initiated by adding 1 mM ATP. At the indicated times, the reaction was terminated by adding 2× Laemmli sample buffer (62.5 mM Tris-HCl, pH 6.8, 2% sodium dodecyl sulfate [SDS], 5% 2-mercaptoethanol, 25% glycerol, 0.01% bromophenol blue). The samples were loaded onto a 7.5% SDS polyacrylamide gel containing 50 μM Phos-tag acrylamide and 0.2 mM MnCl_2_ and electrophoresed at room temperature under a constant voltage (100 V). Phosphorylated proteins interact with the Phos-tag manganese complex, slowing mobility compared with that of nonphosphorylated proteins. Proteins were visualized by staining with Coomassie Brilliant Blue G-250 (0.04% CBB G-250, 3.5% perchloric acid). Gel images were acquired using LuminoGraph I (ATTO, Tokyo, Japan) and quantified using ImageJ.^33^

## Abbreviations

heme: iron protoporphyrin IX
*Af*GcHK: globin-coupled histidine kinase from *Anaeromyxobacter* sp. Fw109-5 or Fe-*Af*GcHK
Co-*Af*GcHK: cobalt-substituted *Af*GcHK
ICP-OES: inductively coupled plasma optical emission spectroscopy
CD: circular dichroism
6cLS: 6-coordinated low-spin
5cHS: 5-coordinated high-spin
5cLS: 5-coordinated low-spin
Mb: myoglobin
Hb: hemoglobin
CoHRP: cobalt-substituted horseradish peroxidase
CoMb: cobalt-substituted myoglobin
CoHb: cobalt-substituted hemoglobin
